# Bronchial Thermoplasty Induced Changes in Blood Transcriptome Profile of Patients with Severe Asthma

**DOI:** 10.3390/ijms27073283

**Published:** 2026-04-04

**Authors:** Sofi M. Vassileva, Jelle M. Blankestijn, Annika W. M. Goorsenberg, Shahriyar Shahbazi Khamas, Stefania Principe, Mahmoud I. Abdel-Aziz, Abilash Ravi, Lizan D. Bloemsma, Els J. M. Weersink, Dirk-Jan Slebos, Pallav L. Shah, Jouke T. Annema, Anke-Hilse Maitland-Van der Zee, Peter I. Bonta

**Affiliations:** 1 Department of Pulmonary Medicine, Amsterdam University Medical Centers, 1105 AZ Amsterdam, The Netherlands; s.vassileva@amsterdamumc.nl (S.M.V.); j.m.blankestijn@amsterdamumc.nl (J.M.B.); a.w.goorsenberg@amsterdamumc.nl (A.W.M.G.); s.shahbazikhamas@amsterdamumc.nl (S.S.K.); s.p.principe@amsterdamumc.nl (S.P.); e.j.weersink@amsterdamumc.nl (E.J.M.W.); j.t.annema@amsterdamumc.nl (J.T.A.); a.h.maitland-van.der.zee@umcg.nl (A.-H.M.-V.d.Z.); 2 Department of Genetics, University Medical Center Groningen, 9713 GZ Groningen, The Netherlands; 3 PulmoScience Lab, Leiden University Medical Center, 2333 ZA Leiden, The Netherlands; 4 Epidemiology & Data Science, Amsterdam University Medical Centers, 1105 AZ Amsterdam, The Netherlands; l.d.bloemsma@amsterdamumc.nl; 5 Groningen Research Institute for Asthma and COPD, University Medical Center Groningen, 9713 GZ Groningen, The Netherlands; d.j.slebos@umcg.nl; 6 National Heart and Lung Institute, Imperial College London, London SW3 6LY, UK; pallav.shah@imperial.ac.uk

**Keywords:** severe asthma, systemic gene expression, transcriptome, bronchial thermoplasty

## Abstract

Bronchial thermoplasty (BT) is a non-pharmacological treatment for severe asthma. The working mechanism and response determinants of BT remain partly unknown. This study aims to investigate whether a systemic transcriptomic response to BT can be detected and contextualized against a control cohort. Whole blood was collected at baseline and six months after BT from severe asthma patients (*n* = 31) and a control cohort (*n* = 126). RNA was isolated and sequenced. The following comparisons were made: before and after BT, responders and non-responders, and severe asthma (at baseline) versus controls. Differentially expressed genes were identified across 179 samples using DESeq2. Pathway enrichment was investigated using gene set enrichment and overrepresentation analyses. Following BT, pathways related to nervous system development, ion channel activity, muscle tissue development, and cilia function were downregulated. In responders specifically, gene sets involved in nervous system and muscle development were downregulated. Compared with the control cohort, pathways related to nervous system development and ion channel activity were upregulated in the severe asthma cohort at baseline. In conclusion, systemic blood-derived transcriptomic changes can be detected in severe asthma patients six months after BT and may provide insight into BT mechanisms and its responder profile.

## 1. Introduction

Severe asthma is a chronic and debilitating condition that significantly impacts quality of life and often proves refractory to conventional treatments. Bronchial thermoplasty (BT) is a non-pharmacological treatment for severe asthma and aims to target airway remodeling. In three sessions, the bronchial tree’s middle- and large-sized airways of >2–3 mm are treated by heating the airway wall to a temperature of 65 °C [1]. Clinical trials have shown that BT treatment results in an improvement in asthma-related quality of life and a reduction in asthma exacerbations, with long-term follow-up data confirming the durability of these effects for up to 10–15 years post-treatment [2,3,4,5,6,7].

In current guidelines, BT is recommended for severe asthma patients who are non-responders to any type-2-targeted therapy or patients who have type-2 low asthma [8]. The precise biological mechanisms driving BT efficacy and the optimal responder profile remain incompletely understood. Elucidating these mechanisms is critical for refining clinical indications and optimizing patient selection in an era of personalized medicine. The most widely shown effect of BT is the reduction in airway smooth muscle (ASM) in biopsies of the treated airways [7,9,10,11]. However, the correlation between the extent of ASM reduction and clinical outcomes is inconsistent, suggesting that alternative mechanisms contribute to therapeutic success. In addition to a reduction in ASM, a reduction in nerves and/or denervation, changes in extracellular matrix components and the epithelium, and an improvement in epithelial integrity have been observed, pointing towards a broad modulation of the airway wall components [9,11,12,13,14].

Compared to biologics for severe asthma, which have established biomarkers to predict response, there is currently limited evidence for biomarkers as predictors for BT response. Based on results from the TASMA trial (Unravelling Targets of Therapy in Bronchial Thermoplasty in Severe Asthma), higher blood eosinophils and IgE levels were associated with the clinical response based on an improvement in asthma-related quality of life and control [7].

To gain deeper biological insight into the mechanisms of BT, transcriptomic profiling can be used to characterize the gene expression signatures linked to treatment effects. Earlier studies investigating transcriptomic changes in the airways found alterations in pathways involving tissue remodeling, metabolism, inflammation, and neurophysiological function [15,16,17,18]. Most of the performed studies have used the transcriptome in airway brushes or biopsies. Whole blood transcriptomics can offer insight into systemic responses that may not be apparent from localized airway analyses alone. To date, systemic blood-derived transcriptomic profiling in the context of BT has received relatively little investigation. This may provide complementary information on the working mechanism of BT and, more importantly, contribute to improved understanding of the responder profile.

The current study aimed to investigate whether the systemic blood-derived transcriptomic response to BT can be detected and to characterize how this response differs between clinical responders and non-responders. The secondary aim was to contextualize the treatment-associated BT response against a reference profile derived from a general-population, non-respiratory-disease control cohort.

## 2. Results

After the exclusion of samples with low counts and reads, a total of 179 samples with blood transcriptome data were available. The severe asthma cohort contributed 52 samples (29 pre- and 23 post-BT) and the control cohort contributed a total of 126 samples. A total of 42 paired samples (paired for baseline and post-BT timepoint) for 21 severe asthma patients were available, eight samples at baseline and two samples post-BT. Table 1 shows the baseline characteristics for both groups. Compared to the control cohort, the severe asthma cohort had a higher proportion of females (74% versus 52%, *p* < 0.001), higher BMI (28 kg/m^2^ versus 26 kg/m^2^, *p* = 0.02) and there was a lower proportion of current smokers (0% versus 9%).

Principal component analysis (PCA) of the different comparisons showed no clear clustering of samples according to (sub)group (Supplemental Table S1).

### 2.1. Transcriptomic Expression of Severe Asthma Before and After BT

**Figure 1:**
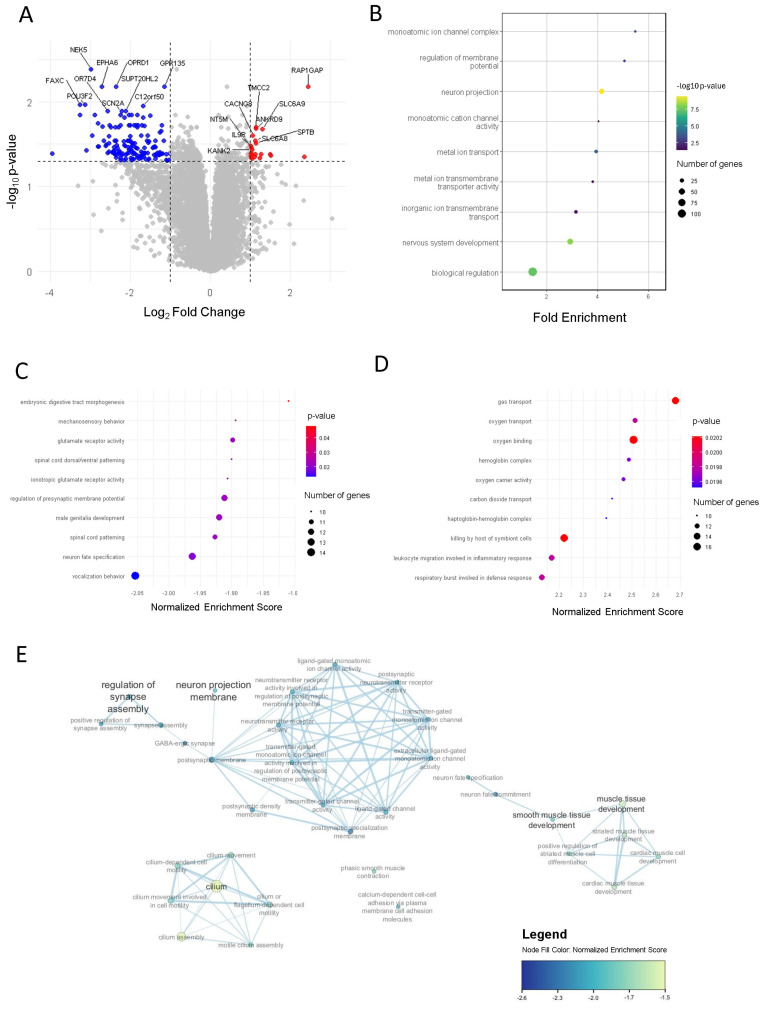
(**A**) Volcano plot of DEGs between pre- and post-BT samples (red = upregulated genes post-BT, blue = downregulated genes post-BT, grey = non-significant genes); (**B**) dotplot of the top 10 downregulated gene sets in post-BT samples compared to pre-BT from ORA; (**C**) dotplot of the top 10 downregulated gene sets in post-BT samples compared to pre-BT from GSEA; (**D**) dotplot of the top 10 upregulated gene sets in post-BT samples compared to pre-BT; (**E**) enrichment plot displaying the biologically relevant gene sets.

Comparative (unpaired) analysis of transcriptomic profiles of patients pre- and post-BT treatment revealed 171 differentially expressed genes (adjusted *p*-value < 0.05, Supplementary Table S1). After BT, a total of 149 genes were downregulated and 22 genes were upregulated (Figure 1A). The top 10 upregulated and downregulated genes are shown in Supplementary Table S2. 

GSEA identified 387 gene sets (Supplementary Table S3). ORA identified 119 gene sets that were downregulated after BT (Supplementary Table S4). The top 10 gene sets for each analysis are displayed in Figure 1B–D. Biologically relevant sets resulting from GSEA that were downregulated after BT are related to nervous system development, ion channel activity, muscle tissue development and cilium function (Figure 1E). Sets upregulated after BT are involved in immune defense, erythrocyte development and hemoglobin function.

The identified gene sets resulting from ORA are related to nervous system development and ion channel activity (Figure 1B). Two sets related to hemoglobin function were found to be upregulated after BT.

Paired analysis of pre- and post-BT samples did not result in significant DEGs, nor did GSEA identify any differential gene sets for this comparison.

### 2.2. Transcriptomic Expression of BT Responders and Non-Responders

**Figure 2:**
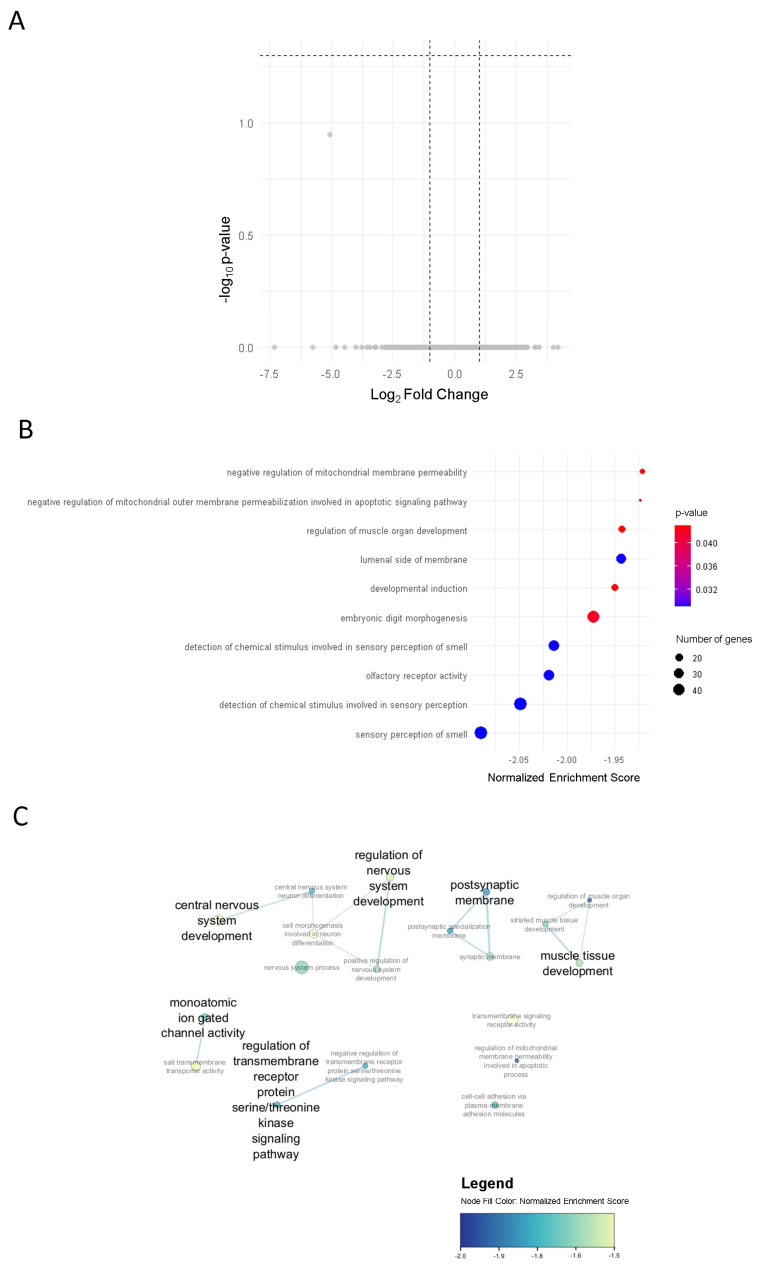
(**A**) Volcano plot showing non-significant DEGs between responders and non-responders post-BT (grey dots = non-significant genes); (**B**) dotplot of the top 10 downregulated gene sets in responders compared to non-responders based on GSEA; (**C**) enrichment plot displaying the biologically relevant gene sets.

In addition to pre- and post-BT analysis, we performed differential gene expression analysis of responders compared to non-responders at baseline (13 responders and 16 non-responders) and post-BT (11 responders and 12 non-responders), separately. This resulted in no significant DEGs (Figure 2A). GSEA of the ranked list of genes from responders and non-responders post-BT resulted in 93 sets which were downregulated in responders (Figure 2B). In line with pre- and post-BT analysis, these sets were related to nervous system development and muscle tissue development (Figure 2C).

### 2.3. Transcriptomic Expression of Severe Asthma Compared to Control Cohort

**Figure 3:**
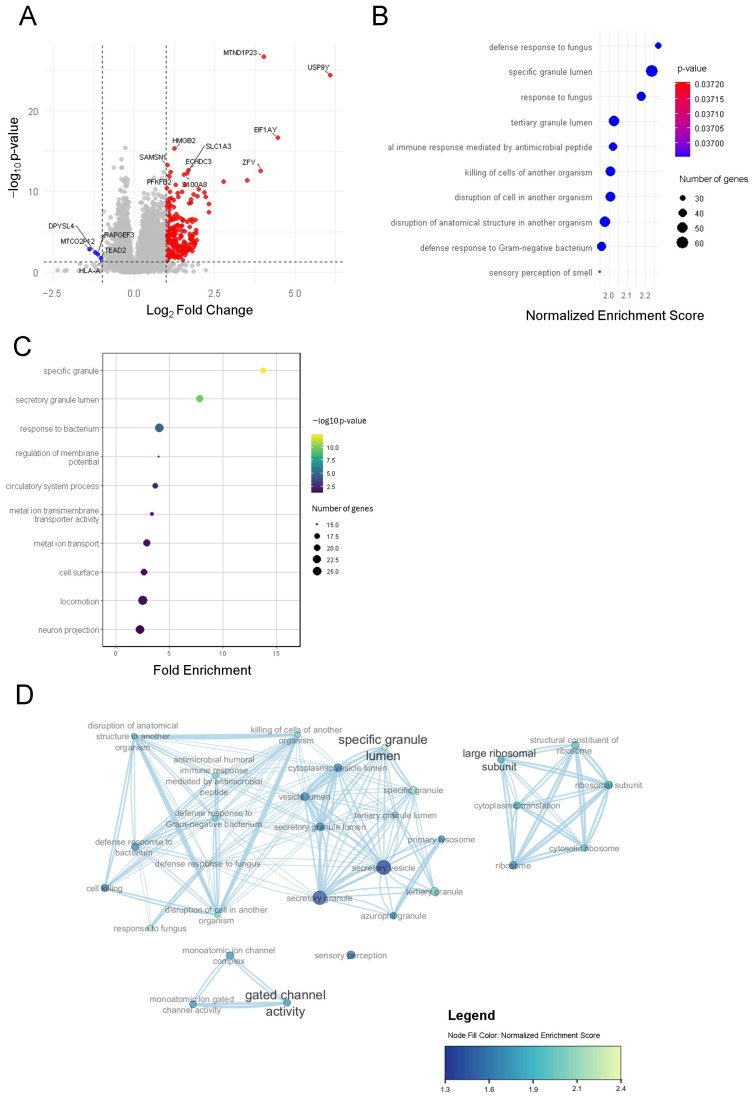
(**A**) Volcano plot of DEGs between asthma and control cohort (red = upregulated in severe asthma, blue = downregulated in severe asthma, grey = non-significant genes); (**B**) dotplot of the top 10 upregulated gene sets in severe asthma compared to control based on GSEA; (**C**) dotplot of the top 10 upregulated gene sets in severe asthma compared to control based on ORA; (**D**) enrichment plot displaying the biologically relevant gene sets.

Differential gene expression analysis of the severe asthma transcriptome at baseline and the control transcriptome resulted in 216 DEGs (Supplementary Table S5). Out of the 216 genes, 211 were upregulated and five were downregulated in severe asthma compared to the control cohort (Figure 3A). GSEA resulted in 30 gene sets that were upregulated in severe asthma compared to control (Supplementary Table S6). ORA revealed 211 gene sets that were upregulated in asthma (Supplementary Table S7). The biologically relevant gene sets in GSEA play a role in immune defense, neutrophil granule biology, protein synthesis and ion channel activity (Figure 3B,D). ORA-identified gene sets are related to four major functions: immune defense, protein synthesis, ion channel activity and nervous system development (Figure 3C).

### 2.4. Overlapping Sets

**Figure 4:**
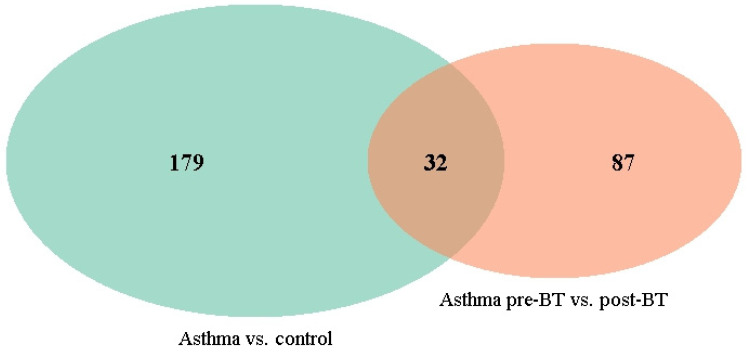
Venn diagram showing the overlapping gene sets (derived from ORA) for severe asthma vs. control cohort comparison and severe asthma pre- and post-BT treatment comparison.

To identify gene sets consistently implicated across conditions, we examined the overlap of GSEA- and ORA-identified gene sets among the three comparisons. The overlap of the ORA gene sets between (1) severe asthma and control cohort, and (2) severe asthma before and after BT, resulted in a total of 33 overlapping gene sets (Figure 4, Table 2). These gene sets are related to nervous system development and ion channel activity.

**Figure 5:**
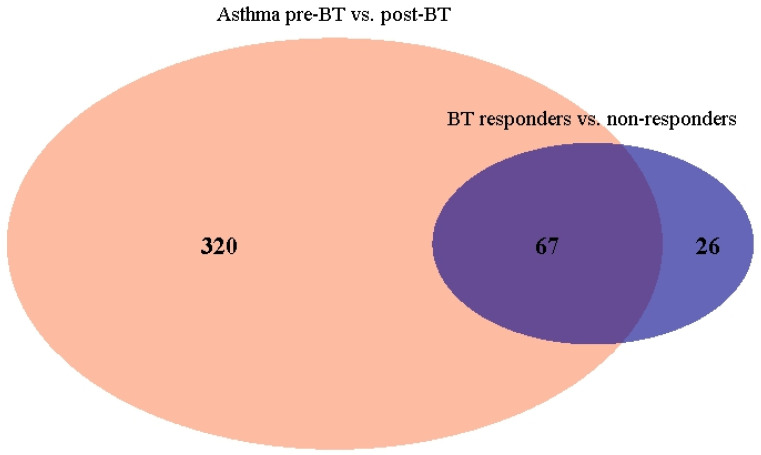
Venn diagram showing overlapping gene sets (derived from GSEA) for severe asthma patients for pre- and post-BT treatment comparison and BT responders vs. non-responders (post-BT) comparison.

The overlap of the GSEA gene sets between (1) severe asthma before and after BT and (2) BT responders versus non-responders, resulted in 67 overlapping sets. The identified gene sets are related to nervous system development, ion channel activity and muscle tissue development (Figure 5, Table 3).

### 2.5. Overlapping Genes

The overlap between the significant differentially expressed genes of the pre- versus post-BT and severe asthma versus the control cohort comparisons was investigated. A total of 51 genes were identified (Supplementary Table S8), including G protein-coupled receptor class C group 5 member A (*GPRC5A*), phospholipase C epsilon 1 (*PLCE1*) and Laminin subunit beta 1 (*LAMB1*).

## 3. Discussion

To our knowledge, this is the first study to characterize the longitudinal systemic transcriptomic effects of BT in patients with severe asthma. Our results showed differences in blood transcriptomic profiles of severe asthma patients undergoing BT. A downregulation of pathways related to nervous system development, muscle tissue development, ion channel activity, and cilium function was observed after BT. Second, overlapping pathways involved in nervous system and muscle tissue development were downregulated in responders. Third, comparison with a control cohort showed that gene sets related to nervous system development and ion channel activity were upregulated in severe asthma. These transcriptional changes in the blood might reflect downstream systemic consequences of previously described local effects of BT on asthmatic airways.

A downregulation of pathways related to the nervous system and smooth muscle was found six months after patients had undergone BT. There is widespread histologic evidence that BT affects the smooth muscle and nervous system in the airways. Most studies demonstrate that BT impacts on airway remodeling by reducing ASM [3,7,10,19]. Although the supporting literature is more limited, several studies have demonstrated that BT might impact the nervous system in the airways as well. Histologically, a decrease in nerves and neuroendocrine cells in the airways has been observed [9,13,19]. Furthermore, systemic transcriptional changes before and after BT have demonstrated a significant decrease in pathways associated with neuron homeostasis [20]. In line with a mechanistic role for denervation in the treatment of obstructive airway diseases, the AIRFLOW-2 trial applied a bronchoscopic intervention specifically aimed at ablating the bronchial branches of the vagus nerve along the central airways and demonstrated a reduction in exacerbations in COPD patients [21]. The found decrease in expression pathways related to the nervous system and muscle tissue possibly mirrors the local effects of BT on the airways. Importantly, as we show that these gene sets were specifically downregulated in responders compared to non-responders of BT, this might imply that the beneficial effects of BT are achieved by affecting the nerves and smooth muscle compartments in the airways.

The comparison between the severe asthma cohort and the control cohort revealed that sets related to the nervous system and muscle tissue were upregulated in the severe asthma cohort. Our finding that the nervous system and muscle tissue-related gene expression decreases after BT directs us towards a possible normalization of expression, as this expression is in line with the control cohort.

We identified a downregulation of pathways related to ion channel activity following BT. Although BT is not currently known to modulate ion channel function, and no published evidence directly links BT to ion channel regulation, these findings raise the possibility that ion transport mechanisms may contribute to the airway’s response to treatment. Ion channels play a central role in the regulation of intracellular Ca^2+^ dynamics and airway smooth muscle tone, thereby raising the possibility that the observed downregulation of ion channel gene expression may be associated with the changes in airway remodeling following BT [22,23,24]. Furthermore, the involvement of ion channel activity, particularly given its role in sustaining inflammation and as a risk factor for childhood asthma when specific genetic variants are present, suggests a remodeling-associated signaling route that has not been described before [22,25].

While further mechanistic studies are needed, our results highlight ion channels as a potential novel therapeutic target of BT in severe asthma.

Our results demonstrated that pathways associated with cilium function are downregulated post-BT. The direction of this result was unanticipated, given the well-established role of impaired ciliary function in the development and persistence of asthma [26,27]. Ciliary dysfunction, leading to poor antigen clearance, has been shown to result in airway epithelial damage and the activation of oxidative pathways, and could thereby predispose individuals to the development of asthma [28]. Furthermore, the downregulation of ciliary genes in the airway compartment has been associated with specific inflammatory phenotypes [29,30]. The precise mechanisms underlying the ciliary downregulation remain unknown. We hypothesize that the observed downregulation may reflect the physical effects of BT, which induces epithelial shedding and alters cellular composition, rather than a functional impairment of the immune response [14,31,32].

In our dataset, post-BT treatment blood transcriptomes showed a higher expression of gene sets involved in immune defense, erythrocyte development and hemoglobin function. Allergen-challenge experiments in sensitized humans and mice have reported transient reductions in circulating erythrocytes and hemoglobin [33,34]. One potential explanation is that reduced bronchial hyperreactivity leads to a decreased migration of erythrocytes and immune cells into the lung tissue. Consequently, more of these cells remain in circulation, potentially accounting for their increased expression in the blood. However, it should be noted that clinical studies on BT have reported conflicting results regarding changes in hyperreactivity [31,35]. These findings remain speculative and should be regarded as hypothesis-generating.

The set of 51 overlapping genes, shared between the pre- and post-BT comparison and the asthma compared to control cohort, includes several genes that have been previously described in the context of asthma. *GPRC5A* encodes for a G protein-coupled receptor involved in the interaction between retinoid acid and G protein signaling pathways. In line with our findings, this gene has been found to be upregulated in the blood and bronchial epithelium of asthma patients [36,37]. Furthermore, a potential role in airway smooth muscle remodeling has also been speculated, strengthening the therapeutic link between BT and airway remodeling [38]. *PLCE1* encodes for an enzyme that belongs to the phospholipase family. A large-scale genome-wide association study has linked a genetic variation in *PLCE1* to a high prevalence of asthma [39]. Mechanistically, PLCE1 is thought to play a central role in the pathogenesis of asthma by stimulating inflammatory cytokine production by bronchial epithelial cells [40]. Laminin beta 1 is the product of *LAMB1* transcription and belongs to the family of extracellular matrix glycoproteins. Research on laminin shows evidence that it plays a role in promoting airway smooth muscle remodeling and airway hyperreactivity [41,42,43]. In our cohort, the expression of these three genes was upregulated in patients with severe asthma compared with controls and showed reduced expression after treatment with BT. Consistent with prior studies highlighting the roles of these genes in asthma pathophysiology, which can be detected by blood-derived transcriptomic profiling, our finding suggests that BT may partially exert its therapeutic effect by reducing the expression these genes.

We observed minimal overlap between the 51 genes identified in this study and those reported in previous transcriptomic investigation of BT. At the individual gene level, only a single gene (*SHISA9*) was shared with work from Liao et al. Pathway analysis identified alterations in neurophysiological processes following BT. While gene-level concordance is minimal, the consistency observed at the pathway level supports that BT exerts its effects on airway neuronal biology. Several factors may account for the limited gene-level overlap. Our study examines systemic transcriptional responses, whereas most prior work has focused on airway-derived samples. Furthermore, bulk transcriptomic data are highly sensitive to differences in cellular composition. Variation between systemic and airway compartments may contribute to the discordance between expression profiles. To further elucidate the cellular drivers of these transcriptional changes, deconvolution approaches may help clarify the cellular origins of these signals. In line with this, prior work in airway brushes from the TASMA severe asthma cohort has demonstrated an expanded basal cell population in BT-treated airways compared to untreated airways, potentially contributing to clinical improvement [32]. A key strength of this study is that prior to sampling, patients did not receive a pulse of oral corticosteroids. Typically, patients undergoing BT are pre-treated with oral corticosteroids as part of standard clinical practice. The absence of corticosteroid exposure minimizes possible confounding related to acute steroid effects. Using both GSEA and ORA strengthens our analysis by combining complementary approaches to pathway enrichment, reducing method-specific bias and increasing confidence in the identified biological processes. Lastly, the incorporation of a control cohort further strengthens the design by allowing for differentiation between BT treatment-related changes and background variability.

Several limitations should be acknowledged. The total sample size of the severe asthma cohort was modest, which contributed to a reduced statistical power and potentially to the absence of detectable differences between timepoints in the paired analysis. Although sampling at six months after BT limits our ability to capture early, acute systemic treatment effects, it enables the assessment of long-term molecular changes that coincide with the delayed clinical response typically observed 6–12 months after BT. Indeed, we identified multiple pathways that align with previously described mechanisms of BT and recognized aspects of asthma pathogenesis, suggesting that these pathways could play a role in the disease pathophysiology.

In conclusion, this study demonstrates systemic changes in gene sets that could well reflect known local BT effects on the nerves and muscle layer in the airways. Interestingly, these sets were shown to be especially downregulated in responders when measured after BT. This suggests that effectively targeting these mechanisms may be particularly important for achieving a treatment response. Collectively, these results support the value of systemic transcriptomic profiling in advancing our understanding of how BT exerts its therapeutic effects. Future research is needed to validate the current findings in the airways. The integration of the appropriate minimally invasive omics strategies can strengthen the understanding of biologically relevant disease mechanisms and improve response prediction across patient populations.

## 4. Materials and Methods

### 4.1. Subjects and Design: Severe Asthma Cohort

The current study forms a sub-study of the TASMA trial (Unravelling Targets of Therapy in Bronchial Thermoplasty in Severe Asthma, ClinicalTrials.gov NCT0222539). This trial enrolled patients with severe asthma eligible for bronchial thermoplasty in the Netherlands and United Kingdom. Patients were included when they fulfilled severe asthma criteria based on the definition of the World Health Organization and Innovative Medicines Initiative [44,45]. The inclusion and exclusion criteria have been previously described in detail in the original trial publication [7]. The asthma medication for all patients was kept unchanged throughout the study visits until 6 months after BT. Ethical approval was provided by the local ethics committee of the Amsterdam UMC (2013_328).

For all patients, whole blood samples were collected at baseline and 6 months after BT. Furthermore, basic demographics, white blood cell and differential counts, exhaled FeNO, quality of life questionnaires (Asthma Quality of Life Questionnaire (AQLQ) and Asthma Control Questionnaire-6 (ACQ-6) and pulmonary functions tests were collected both before and after BT.

Clinical responders to BT were defined as an improvement in asthma-related quality of life measured by an increase equal to at least 0.5 points in AQLQ score after BT. Patients who did not fulfill this criterium were defined as non-responders.

#### 4.1.1. Bronchial Thermoplasty

BT was performed in three sessions using the Alair system (Boston Scientific, Marlborough, MA, USA) while patients were deeply sedated, as described previously [7].

#### 4.1.2. Subjects and Design: Control Cohort

The control cohort was included as a reference population to act as a biological reference frame for the interpretation of blood-derived BT treatment-associated gene expression changes. Participants from the ongoing Dutch P4O2 PARASOL cohort (Prevention of And Risk fActorS for chronic disease: an Observational study in North HoLland) were included as a control cohort. Written informed consent was obtained prior to inclusion. Inclusion criteria were ages 40–55 years, residing in (the vicinity of) Amsterdam or Hoorn, the ability to provide informed consent and understanding of the Dutch language. For the current study, patients with a known respiratory disease were excluded. Ethical approval was provided by the local ethics committee of the Amsterdam UMC (2023.0179). For PARASOL participants, fasting blood samples were collected during the baseline study visit.

### 4.2. Sample Preparation, RNA Isolation and Sequencing

Whole blood was collected in PAXgene tubes (Qiagen, Venlo, The Netherlands) from 31 patients from the severe cohort (a total of 52 samples) and 126 participants from the control cohort. RNA was extracted from whole blood samples at the Core Facility Genomics of Amsterdam UMC using the QIAcube and the PAXgene blood kit (Qiagen, Venlo, The Netherlands). Only samples with an RIN (RNA Integrity Number) of 6 or higher were included in the final analysis. Library preparation was performed with the KAPA mRNA HyperPrep (Roche, Basel, Switzerland) and thereafter sequenced using the NovaSeq Xplus PE150 platform and NovaSeq™ X Series 10B Rgt Kit (300Cy) (Illumina, Cambridge, UK).

### 4.3. Read Processing

The quality of paired-end sequencing reads was evaluated using FastQC (v0.12.1). Adapter sequences, low-quality reads and low-quality bases at read termini were trimmed using Trim Galore (v0.10.6), a tool that integrates Cutadapt (v4.3) and FastQC. Reads were subsequently mapped and quantified using Salmon (v1.10.1), with the human GRCh38.p14 reference genome and gene annotations obtained from Ensembl (vGRCh38.109).

### 4.4. Statistical Analysis

Statistical analysis was performed in R (version 4.3.2) and RStudio (version 2024.12.1) [46]. Patient characteristics were described as mean (standard deviation), median (IQR) or *n* (%). Baseline characteristics between two groups were compared using independent *t*-test (numerical variables), chi-squared test (categorical variables) and Fisher’s exact test (categorical variables with low counts).

Four samples were rejected due to having fewer than 500,000 read counts, leaving 179 samples ready for statistical analysis. Lowly abundant genes were removed from the data (<100 reads or present in fewer than 20% of samples in either dataset). Data were normalized using size factor normalization with the DESeq2 package [47] and log_2_ transformed after addition of a pseudocount.

DESeq2 analysis was applied for unbiased identification of differentially expressed genes (DEGs), where models were adjusted for sex, body mass index (BMI) and smoking status. DEGs were tested for the following comparisons: (1) severe asthma before and after BT, (2) severe asthma responders to BT and non-responders, (3) severe asthma cohort and control cohort. For the first comparison, both paired and unpaired DESeq2 analyses were performed. The latter was done to account for missing paired samples and maximize the sample size. Significant DEGs were defined as those with an adjusted *p* < 0.05, using the Benjamini–Hochberg correction for multiple testing.

Pathway analysis was performed using two statistical techniques to ensure robust findings: gene set enrichment analysis (GSEA) from the clusterProfiler package (v4.10.1) [48] and over representation analysis (ORA) using the online platform g:Profiler [49]. A ranked gene set list based on the DESeq2 fold change was used as input for the GSEA. For the ORA, only significant DEGs were used as input.

### 4.5. Visualization of Genes and Gene Sets

To capture variation in the subgroups for the different comparisons, a principal component analysis (PCA) was performed. The input data for the PCA were the normalized transcriptomic data.

To identify genes involved in both the pre- versus post-BT treatment comparison and the severe asthma versus control cohort, the overlap among significantly differentially expressed genes between these two comparisons was examined and listed.

Biologically relevant gene sets (defined as genes that are related to functions that are known to play a role in asthma pathogenesis) resulting from the GSEA were categorized by overlapping themes. To maintain figure readability, the top 10 gene sets per category were selected based on their normalized enrichment score (NES). Cytoscape (version 3.10.4) was used to visualize these gene sets in an enrichment plot, which displays the relationships among all selected gene sets (each line represents overlapping genes between the sets).

To identify pathways that are consistently implicated across comparisons and conditions, overlapping gene sets in GSEA (for asthma vs. control cohort comparison and patients for pre- and post-BT treatment comparison) and ORA (asthma patients for pre- and post-BT treatment comparison and responder vs. non-responder (post-BT) comparison) were selected and displayed in a Venn diagram.

## Figures and Tables

**Table 1:** Patient characteristics at baseline.

	Severe Asthma Cohort (*n* = 31)	Control Cohort (*n* = 126)	*p*-Value
Sex, female, *n* (%)	23 (74)	68 (52)	<0.001
Age in years, mean (SD)	44.8 (13.4)	48.0 (4.6)	0.20
BMI, kg/m^2^, mean (SD)	28.1 (4.6)	25.8 (4.5)	0.02
Smoking status			0.04
Current, *n* (%)	0	11 (9)	
Former, *n* (%)	20 (64)	65 (55)	
Never, *n* (%)	11 (36)	42 (36)	
ACQ-6 score, mean (SD)	2.8 (0.8)		
AQLQ score, mean (SD)	4.1 (0.9)		
Blood eosinophils, 10^9^/L, median (IQR)	0.17 [0.09, 0.30]		
Positive skin prick test, *n* (%)	23 (74)		
Pre-BD FEV1, % predicted, mean (SD)	84.9 (21.5)		
Post-BD FEV1, % predicted, mean (SD)	96.9 (20.1)		
FEV1 reversibility, % predicted, median (IQR)	9.50 [4.25, 13.75]		
FVC, % predicted, mean (SD)	96.9 (19.5)		
PC20, mg/mL, median (IQR)	0.25 [0.03, 2.00]		
FeNO, ppb, median (IQR)	18.0 [13.0, 49.5]		
ICS dose, µg/day fluticasone equivalent, median (IQR)	1000.0 [1000.0, 1375.0]		
Maintenance OCS, *n* (%)	9 (29)		
OCS dose, mg, mean (SD)	11.9 (5.3)		
Omalizumab use, *n* (%)	4 (12.9)		

**Table 2:** List of 32 overlapping gene sets between severe asthma vs. control cohort and severe asthma pre- versus post-BT comparison.

Term ID	Term Name	*p*-Value (Pre- vs. Post-BT)	Enrichment Score (Pre- vs. Post-BT)	*p*-Value (Asthma vs. Healthy)	Enrichment Score (Asthma vs. Healthy)
GO:0001508	action potential	0.001663738	8.662973814	0.020537049	6.433904529
GO:0005886	plasma membrane	1.94117 × 10^−7^	1.944269023	0.002174179	1.568102933
GO:0007154	cell communication	3.20458 × 10^−6^	1.822668497	0.000208772	1.616892895
GO:0007275	multicellular organism development	9.61712 × 10^−8^	2.171495963	0.011385186	1.664772608
GO:0007399	nervous system development	6.88502 × 10^−9^	2.924622325	0.000130488	2.219307229
GO:0007417	central nervous system development	0.002393448	3.276852175	0.009439282	2.781354895
GO:0022008	neurogenesis	7.58038 × 10^−5^	2.89798459	0.009345964	2.291162689
GO:0023052	signaling	6.10417 × 10^−5^	1.753426677	0.000172878	1.623097396
GO:0030001	metal ion transport	0.000117987	3.937418072	0.018221043	2.924281258
GO:0030154	cell differentiation	0.008112466	1.828351071	0.013118253	1.690444653
GO:0032501	multicellular organismal process	1.33221 × 10^−6^	1.763671988	3.67848 × 10^−5^	1.59534432
GO:0032502	developmental process	1.17593 × 10^−6^	1.844317263	0.001153322	1.576157699
GO:0034702	monoatomic ion channel complex	0.000707484	5.479261153	0.016534034	4.012136582
GO:0034703	cation channel complex	0.003159218	6.611641791	0.00612912	5.379234973
GO:0042391	regulation of membrane potential	0.001178455	5.060757143	0.009242611	4.027043592
GO:0043005	neuron projection	2.28283 × 10^−10^	4.147524371	0.044834513	2.233077249
GO:0044309	neuron spine	0.042140648	6.32260827	0.047606114	5.291050793
GO:0045202	synapse	6.38843 × 10^−8^	3.375312626	0.034242185	2.10807857
GO:0046873	metal ion transmembrane transporter activity	0.018755259	3.807841435	0.015495418	3.370120682
GO:0048468	cell development	0.002441656	2.187497947	0.005947943	1.966660973
GO:0048731	system development	6.49223 × 10^−8^	2.32836569	0.001365417	1.820267422
GO:0048856	anatomical structure development	4.32854 × 10^−8^	1.987702513	0.005117344	1.578764041
GO:0048869	cellular developmental process	0.008166023	1.827939094	0.013216041	1.69006375
GO:0050789	regulation of biological process	6.95074 × 10^−7^	1.436026175	9.18189 × 10^−6^	1.355579827
GO:0050794	regulation of cellular process	9.19378 × 10^−5^	1.399754275	1.49874 × 10^−6^	1.389542415
GO:0065007	biological regulation	3.6396 × 10^−8^	1.442129414	7.57784 × 10^−6^	1.34123126
GO:0071944	cell periphery	8.91043 × 10^−7^	1.849009639	0.000163857	1.601820403
GO:1902495	transmembrane transporter complex	0.014602173	4.047943954	0.019714114	3.458079625
GO:1990351	transporter complex	0.024835096	3.829136172	0.035486732	3.271156402
REAC:R-HSA-112316	neuronal System	3.57075 × 10^−8^	6.313253012	0.049769766	3.190952588
REAC:R-HSA-5576892	phase 0—rapid depolarization	0.039039235	16.57228916	0.000466466	17.05165289
REAC:R-HSA-9675108	nervous system development	0.03360585	3.267775327	0.001424798	3.202188337

**Table 3:** List of 67 overlapping gene sets from severe asthma versus control cohort comparison and BT responders versus non-responders comparison.

Term ID	Term Name	*p*-Value (Pre- vs. Post-BT)	Enrichment Score (Pre- vs. Post-BT)	*p*-Value (Responder vs. Non-Responder)	Enrichment Score (Responder vs. Non-Responder)
GO:0001654	eye development	0.012167742	−0.456631052	0.044174687	−0.457384675
GO:0001708	cell fate specification	0.012167742	−0.681636467	0.049329695	−0.645989209
GO:0001822	kidney development	0.012167742	−0.480365302	0.028745998	−0.509737807
GO:0002009	morphogenesis of an epithelium	0.025814294	−0.404538581	0.028745998	−0.454701749
GO:0003002	regionalization	0.012167742	−0.473946679	0.028745998	−0.491450337
GO:0003007	heart morphogenesis	0.047536635	−0.436362854	0.028745998	−0.503482554
GO:0004888	transmembrane signaling receptor activity	0.012167742	−0.453477895	0.028745998	−0.411490261
GO:0004984	olfactory receptor activity	0.012167742	−0.815775871	0.029029477	−0.762042176
GO:0005814	centriole	0.033298352	−0.468824351	0.038599194	−0.520054924
GO:0007156	homophilic cell adhesion via plasma membrane adhesion molecules	0.012167742	−0.583723379	0.028745998	−0.614579361
GO:0007389	pattern specification process	0.012167742	−0.480303955	0.028745998	−0.49750461
GO:0007417	central nervous system development	0.012167742	−0.40176476	0.028745998	−0.396308081
GO:0007420	brain development	0.012167742	−0.425758666	0.036195416	−0.407718275
GO:0007423	sensory organ development	0.012167742	−0.456184343	0.028745998	−0.481675861
GO:0007507	heart development	0.021620244	−0.391091503	0.028745998	−0.439379013
GO:0007600	sensory perception	0.012167742	−0.54319628	0.028745998	−0.499171352
GO:0007606	sensory perception of chemical stimulus	0.012167742	−0.69300814	0.028745998	−0.683441515
GO:0007608	sensory perception of smell	0.012167742	−0.771266822	0.028990109	−0.735921879
GO:0007610	behavior	0.012167742	−0.447562176	0.028745998	−0.442111432
GO:0007611	learning or memory	0.012167742	−0.483395385	0.028745998	−0.502035627
GO:0007612	learning	0.012167742	−0.561243504	0.046991809	−0.541781816
GO:0009593	detection of chemical stimulus	0.012167742	−0.683973729	0.028745998	−0.654886098
GO:0009887	animal organ morphogenesis	0.012167742	−0.409146447	0.028745998	−0.432843106
GO:0014706	striated muscle tissue development	0.02664229	−0.464535448	0.044857712	−0.495116981
GO:0021953	central nervous system neuron differentiation	0.012167742	−0.558378917	0.038599194	−0.54082887
GO:0022839	monoatomic ion gated channel activity	0.012167742	−0.562338823	0.038599194	−0.497758451
GO:0030326	embryonic limb morphogenesis	0.018410013	−0.56684809	0.028745998	−0.62426934
GO:0030425	dendrite	0.012167742	−0.412425168	0.049329695	−0.401990582
GO:0031128	developmental induction	0.024848431	−0.75856895	0.042947488	−0.815920134
GO:0034330	cell junction organization	0.012167742	−0.429993506	0.028745998	−0.426752755
GO:0035107	appendage morphogenesis	0.012167742	−0.540804131	0.028745998	−0.571993762
GO:0035108	limb morphogenesis	0.012167742	−0.540804131	0.028745998	−0.571993762
GO:0035113	embryonic appendage morphogenesis	0.018410013	−0.56684809	0.028745998	−0.62426934
GO:0036477	somatodendritic compartment	0.012167742	−0.411774057	0.036195416	−0.389203451
GO:0038023	signaling receptor activity	0.012167742	−0.420173536	0.028745998	−0.402624713
GO:0043583	ear development	0.012167742	−0.555270019	0.028745998	−0.612070049
GO:0045165	cell fate commitment	0.012167742	−0.516023321	0.028745998	−0.524467543
GO:0045211	postsynaptic membrane	0.012167742	−0.616206696	0.028745998	−0.5446129
GO:0048562	embryonic organ morphogenesis	0.012167742	−0.504567221	0.028745998	−0.56797415
GO:0048568	embryonic organ development	0.023728476	−0.412198595	0.028745998	−0.466630221
GO:0048598	embryonic morphogenesis	0.021620244	−0.395922175	0.028745998	−0.492074258
GO:0048667	cell morphogenesis involved in neuron differentiation	0.012167742	−0.462737373	0.049329695	−0.412109505
GO:0048706	embryonic skeletal system development	0.020460057	−0.579353701	0.040449548	−0.60446373
GO:0048729	tissue morphogenesis	0.021620244	−0.39461548	0.028745998	−0.458505835
GO:0048736	appendage development	0.012167742	−0.503222877	0.038599194	−0.529879334
GO:0048839	inner ear development	0.012167742	−0.578872328	0.028745998	−0.626279716
GO:0050877	nervous system process	0.012167742	−0.482284887	0.028745998	−0.448526354
GO:0050906	detection of stimulus involved in sensory perception	0.012167742	−0.683182351	0.028745998	−0.611229846
GO:0050907	detection of chemical stimulus involved in sensory perception	0.012167742	−0.757977874	0.028990109	−0.723710208
GO:0050911	detection of chemical stimulus involved in sensory perception of smell	0.012167742	−0.809647432	0.029029477	−0.755936074
GO:0051606	detection of stimulus	0.012167742	−0.588346579	0.028745998	−0.564182382
GO:0051960	regulation of nervous system development	0.019805802	−0.418098319	0.043004315	−0.434790623
GO:0051962	positive regulation of nervous system development	0.023806946	−0.436869851	0.044515511	−0.470837953
GO:0060089	molecular transducer activity	0.012167742	−0.420173536	0.028745998	−0.402624713
GO:0060173	limb development	0.012167742	−0.503222877	0.038599194	−0.529879334
GO:0060322	head development	0.012167742	−0.419190684	0.042947488	−0.395999217
GO:0060537	muscle tissue development	0.017277489	−0.430958173	0.028745998	−0.457606318
GO:0060993	kidney morphogenesis	0.018842154	−0.614871267	0.040996956	−0.632101756
GO:0072001	renal system development	0.012167742	−0.476190098	0.028745998	−0.504326546
GO:0072006	nephron development	0.012167742	−0.544638245	0.028745998	−0.566006562
GO:0072009	nephron epithelium development	0.027329987	−0.543290569	0.048403111	−0.599112627
GO:0090596	sensory organ morphogenesis	0.012167742	−0.499199187	0.044857712	−0.491738426
GO:0097060	synaptic membrane	0.012167742	−0.549024991	0.038456459	−0.463315533
GO:0098742	cell–cell adhesion via plasma–membrane adhesion molecules	0.012167742	−0.56849251	0.028745998	−0.522530051
GO:0099634	postsynaptic specialization membrane	0.012167742	−0.698108346	0.048403111	−0.593964624
GO:1901702	salt transmembrane transporter activity	0.012167742	−0.447219553	0.049555967	−0.423141758
GO:2000027	regulation of animal organ morphogenesis	0.025022203	−0.52951177	0.028745998	−0.624789671

## Data Availability

The data are available on request to the corresponding author. The control cohort data are not publicly available due to agreements made by the consortium, that only allow access by each consortium partner to specific data that answers their pre-specified research questions. A request for access to data by organizations outside of the consortium can be submitted to the P4O2 Data Committee (via p4o2@amsterdamumc.nl) and the research will need to be performed in collaboration with one of the P4O2 consortium partners.

## Supplementary Materials

The following supporting information can be downloaded at: https://www.mdpi.com/article/10.3390/ijms27073283/s1.

## Abbreviations

The following abbreviations are used in this manuscript:

ACQ-6	Asthma
AQLQ	Asthma quality of life questionnaire
ASM	Airway smooth muscle
BMI	Body mass index
BT	Bronchial thermoplasty
GPRC5A	G protein-coupled receptor class C group 5 member A
GSEA	Gene set enrichment analysis
LAMB 1	Laminin subunit beta 1
NES	Normalized enrichment score
ORA	Over-representation analysis
PLCE1	Phospholipase C epsilon 1
RNA	Ribonucleic acid
